# A malignant network drives outcome in DIPG/DMG

**DOI:** 10.1093/neuonc/noag183

**Published:** 2026-08-31

**Authors:** Michelle Monje

**Affiliations:** Department of Neurology and Neurological Sciences, Stanford University (M.M.); Howard Hughes Medical Institute, Stanford University (M.M.)

Diffuse midline gliomas (DMG) are lethal cancers that occur in the brainstem, most often the ventral pons (pontine DMG is also called diffuse intrinsic pontine glioma, DIPG), thalamus, and spinal cord. As the name implies, these gliomas are diffusely infiltrative and often spread throughout the nervous system. A groundbreaking new study^1^ from Darren Hargrave’s group at Great Ormond Street Hospital for Children (GOSH) and University College London (UCL) has identified a distributed, functionally connected brain-to-tumor network strongly associated with tumor progression and patient survival in children with DMG.

Preclinical discoveries over the past decade have revealed that DMGs integrate into neural circuits^2^ and that glioma growth^2-9^ and invasion^10^ are driven by circuit activity. Multiple types of neurons influence DMG—including glutamatergic,^2^^,^^3^^,^^5^ GABAergic,^6^ cholinergic,^7^ serotonergic,^8^ and noradrenergic^9^ neurons—through activity-regulated paracrine signaling^3-5^^,^^7^^,^^10^ and through bona fide neuron-to-glioma synapses.^2^^,^^5^^,^^6^ The influence of neurons on DMG occurs at both local^6^ and long-range^2^^,^^7^^,^^8^ scales, indicating brain-wide interactions that appear central to DMG pathophysiology in preclinical models. However, the extent to which this is true in patients, and which neural circuits may be most consistently and importantly involved, remained to be determined.

The study from Darren Hargrave’s group at GOSH and UCL, led by clinician-scientist (MBPhD) students Jai Sidpra and Valentina Lind, defined the brain-wide network of connectivity between specific neural circuits and DMG, which is inversely associated with the overall survival of DMG patients and positively correlated with the circuit-specific patterns of DMG growth. This endeavor required the development of a new computational neuroimaging approach called Tumor Network Mapping, which analyzed pontine and thalamic DMG patient MRI scans and used functional (fMRI) and structural (DTI) connectivity data from 1000 healthy pediatric subjects to compute the patterns of brain-to-DMG tumor connectivity. Both pontine and thalamic DMG map to a conserved brain network that strongly associates with shorter patient survival, regardless of primary tumor location (pontine or thalamic). This prognostic DMG network was identified in a discovery cohort of 125 patients treated at GOSH (106 pontine DMG/DIPG, 19 thalamic DMG) and confirmed in 2 validation cohorts (125 in a multi-center cohort and 38 in a Pediatric Neuro-Oncology Consortium (PNOC) cohort). The DMG network involves motor, cerebellar, limbic, and insular circuitry, with strong functional connectivity of tumor to the motor cortex (M1 and supplementary motor area (SMA)), orbitofrontal (OFC) and insular cortices, limbic system (amygdala, hippocampus, basal ganglia, and anterior cingulate cortex (ACC)), superior temporal gyrus (STG), cerebellum (lobules I-V, VIII, and X), and of course to the pons and thalamus (centromedian, medial pulvinar, and ventral intermediate nuclei).^1^

The neuroanatomical topography of this DMG network mirrors the observed clinical pattern of DMG spread. The authors determined that, over time, DMG exhibits directional growth towards the network, with direct extension of pontine and thalamic DMG along network trajectories. Furthermore, brain parenchymal metastases from spinal DMG tend to occur within the DMG network. Taken together, these findings illustrate that DMG growth and spread maps to a conserved brain network, and connectivity of the tumor with this distributed network is independently and inversely associated with patient overall survival.^1^

Neural circuits mature over the course of postnatal neurodevelopment with respect to synaptic connectivity and developmental myelination. Accordingly, neural circuit activity changes dynamically over childhood and adolescence. To assess spatiotemporal patterns of metabolic indicators of circuit activity within the DMG network, the authors examined [^18^F]FDG-PET imaging of healthy children over the course of postnatal brain development from infancy to age 16. Strikingly, 2 peaks of neurometabolic activity—at 5-6 and 10-11 years of age—were evident within the DMG network, concordant with developmental timepoints of peak DMG incidence.^1^ While correlative, these findings link DMG incidence with neurometabolic circuit activity—consistent with the strong preclinical evidence that neuronal activity drives DMG growth and invasion.^2-8^^,^^10^

Single nucleus transcriptomic studies of primary patient DMG samples from regions of high functional connectivity demonstrate expression of synaptic gene sets, with enrichment in the OPC-like malignant cell state, synaptic scores,^2^ and serotonergic, cholinergic and noradrenergic receptor genes. Particularly notable are strong associations of the expression of neurologin-3 (NLGN3)-responsive genes—a key neuronal activity-regulated signaling pathway in DMG,^3^^,^^4^ serotonin receptor 5HT2A, cholinergic receptor M1, and glutamatergic genes with high functional connectivity.^1^ Concordantly, tumor functional connectivity with cholinergic, serotonergic, and noradrenergic brainstem nuclei strongly contributes to the prognostic DMG network.^1^ These human findings implicate exactly the same mechanisms of long-range neuron-glioma interactions found to drive DMG pathophysiology in preclinical studies.^2-4^^,^^7-9^

The consistent and predictable manner in which DMG integrates into specific neural circuits, the prognostic significance for patient survival, and the weight of mechanistic evidence that the underlying mechanisms of neuron-glioma interactions drive DMG pathophysiology all highlight the therapeutic importance of disrupting this malignant network. Pharmacological strategies are in development to block neuron-glioma signaling and mitigate the structural and functional integration of this tumor into the neural circuits it invades. Non-pharmacological strategies to disconnect the tumor from the DMG network may also play a therapeutic role. In this vein, the authors found that surgical approaches for thalamic DMG biopsy that disrupt the DMG network confer a survival benefit, irrespective of the tumor volume resected.^1^ This raises the possibility that interventions to surgically or otherwise disconnect this brain-to-­tumor network where safe and feasible could be beneficial and should be studied in prospective clinical trials.

This is a landmark study for both the fields of pediatric neuro-oncology and cancer neuroscience. The work provides critical insights into the fundamental biology of this lethal childhood brain cancer, with important prognostic and treatment implications. The data presented identify the brain-wide connectivity pattern of DMG and underscore the critical importance of this malignant network to DMG pathophysiology. Effective therapeutic strategies for DMG will require disconnection—pharmacologically or non-pharmacologically—of these powerfully pathogenic network interactions.
